# COVID-19 vaccines that reduce symptoms but do not block infection need higher coverage and faster rollout to achieve population impact

**DOI:** 10.1038/s41598-021-94719-y

**Published:** 2021-07-30

**Authors:** David A. Swan, Chloe Bracis, Holly Janes, Mia Moore, Laura Matrajt, Daniel B. Reeves, Eileen Burns, Deborah Donnell, Myron S. Cohen, Joshua T. Schiffer, Dobromir Dimitrov

**Affiliations:** 1 Vaccine and Infectious Disease Division, Fred Hutchinson Cancer Research Center, 1100 Fairview Ave N., M2-C200, P.O. Box 19024, Seattle, WA 98109-1024 USA; 2 Université Grenoble Alpes, TIMC-IMAG/BCM, 38000 Grenoble, France; 3 Independent Researcher, Seattle, WA USA; 4 Department of Global Health, University of Washington, Seattle, WA USA; 5 Department of Epidemiology, University of North Carolina at Chapel Hill, Chapel Hill, NC USA; 6 Clinical Research Division, Fred Hutchinson Cancer Research Center, Seattle, WA USA; 7 Department of Medicine, University of Washington, Seattle, WA USA; 8 Department of Applied Mathematics, University of Washington, Seattle, WA USA

**Keywords:** Viral infection, Applied mathematics

## Abstract

Trial results for two COVID-19 vaccines suggest at least 90% efficacy against symptomatic disease (VE_DIS_). It remains unknown whether this efficacy is mediated by lowering SARS-CoV-2 infection susceptibility **(**VE_SUSC_) or development of symptoms after infection (VE_SYMP_). We aim to assess and compare the population impact of vaccines with different efficacy profiles (VE_SYMP_ and VE_SUSC_) satisfying licensure criteria. We developed a mathematical model of SARS-CoV-2 transmission, calibrated to data from King County, Washington. Rollout scenarios starting December 2020 were simulated with combinations of VE_SUSC_ and VE_SYMP_ resulting in up to 100% VE_DIS_. We assumed no reduction of infectivity upon infection conditional on presence of symptoms. Proportions of cumulative infections, hospitalizations and deaths prevented over 1 year from vaccination start are reported. Rollouts of 1 M vaccinations (5000 daily) using vaccines with 50% VE_DIS_ are projected to prevent 23–46% of infections and 31–46% of deaths over 1 year. In comparison, vaccines with 90% VE_DIS_ are projected to prevent 37–64% of infections and 46–64% of deaths over 1 year. In both cases, there is a greater reduction if VE_DIS_ is mediated mostly by VE_SUSC_. The use of a “symptom reducing” vaccine will require twice as many people vaccinated than a “susceptibility reducing” vaccine with the same 90% VE_DIS_ to prevent 50% of the infections and death over 1 year. Delaying the start of the vaccination by 3 months decreases the expected population impact by more than 50%. Vaccines which prevent COVID-19 disease but not SARS-CoV-2 infection, and thereby shift symptomatic infections to asymptomatic infections, will prevent fewer infections and require larger and faster vaccination rollouts to have population impact, compared to vaccines that reduce susceptibility to infection. If uncontrolled transmission across the U.S. continues, then expected vaccination in Spring 2021 will provide only limited benefit.

## Introduction

SARS-CoV-2 has infected more than 95 million people around the world as of January 19, 2021, causing more than 2 million deaths^1^. With the onset of winter in the Northern Hemisphere, cases are surging particularly in Europe and North America. The need for a COVID-19 vaccine is the most urgent public health priority in recent human history^2,3^.

Two mRNA-based vaccines have already demonstrated efficacy greater than 90%^4,5^, two more viral vector vaccines have proven effective^6,7^ with several more vaccine candidates in the final stage of testing. The primary objective in each of these studies is to evaluate vaccine efficacy against virologically-confirmed symptomatic SARS-CoV-2 infection, i.e. COVID-19 disease, denoted VE_DIS_. The COVID-19 endpoint is defined by protocol-specified criteria for signs and symptoms consistent with COVID-19 and a confirmatory SARS-CoV-2 PCR test. Asymptomatic cases are therefore not captured in the primary endpoint of these trials, though seroconversion data will ultimately be available for all participants and potentially used to assess the proportion of asymptomatic infections in the active and placebo arms.

The population impact of a vaccine is not fully captured by its efficacy against disease; several other types of vaccine effects may have equal or greater influence^8^. First, a COVID-19 vaccine may reduce the likelihood of acquiring SARS-CoV-2 upon exposure, i.e. vaccine efficacy on susceptibility (VE_SUSC_)^9,10^. Alternatively, or in combination, a vaccine might reduce the likelihood of symptoms upon infection (VE_SYMP_) and lead to subclinical (asymptomatic) infection with viral shedding that might still allow ongoing transmission. Critically, both VE_SUSC_ and VE_SYMP_ would contribute to observed VE_DIS_. Finally, a vaccine may decrease the infectiousness of individuals who become infected (VE_INF_). A vaccine’s population impact depends on all these vaccine effects.

A specific concern for COVID-19 vaccines is that a vaccine with high efficacy against COVID-19 disease (VE_DIS_) but low efficacy against SARS-CoV-2 infection (VE_SUSC_), would predominantly convert symptomatic infections to asymptomatic infections. Furthermore, if the vaccine does not reduce infectiousness (VE_INF_ = 0), it could in theory lead to increased spread of SARS-CoV-2^11,12^. This is especially relevant in settings such as the US where the testing and diagnosis of infection is primarily symptom-driven; individuals with asymptomatic infection are less likely to be diagnosed and therefore may spread infection more readily than diagnosed and socially-isolated symptomatic individuals. The concern is heightened by considerable evidence pointing to the role that asymptomatic and pre-symptomatic cases play in transmission of SARS-CoV-2, with peak infectiousness typically occurring prior to the onset of symptoms^13–15^. The Pfizer and Moderna vaccines are reported to have ~ 95% VE_DIS_ with VE_SUSC_ and VE_SYMP_ remaining unknown. Unfortunately, VE_INF_ for these vaccines is not estimable from the completed trials.

Mathematical models are useful tools to simulate epidemic dynamics and several models have already projected the population impact of hypothetical COVID-19 vaccines^16–25^. This work has mostly revolved around vaccine prioritization groups^16,17^ and none have specifically assessed the population impact of “symptom averting” vaccines with high VE_DIS_ but low VE_SUSC_ and VE_INF_. As a result, the modeling projections of achieving more than 80% population effectiveness with 40% coverage may be overly optimistic^23^. Moreover, most of the previous work has not considered vaccination rollouts initiated in the middle of epidemic wave which may reduce the overall benefits from the vaccination.

Employing a mathematical model calibrated to King County, WA, we analyze the expected population benefits of a COVID-19 vaccine with demonstrated efficacy in reducing COVID-19 disease under varying combinations of vaccine effects (VE_SUSC_ and VE_SYMP_) and under different rollout timing and population coverage. Our results suggest that the concern for increased transmission due to rollout of COVID-19 vaccines with at least 50% VE_DIS_ that mostly converts symptomatic to asymptomatic infections (low VE_SUSC_ and high VE_SYMP_) is not supported by the evidence. However, we demonstrate that vaccines that do reduce the risk of infection (high VE_SUSC_ and low VE_SYMP_) are expected to have substantially greater population impact. Although focused on King County, WA, we consider an ensemble of plausible epidemic trajectories with variable projections of the timing and magnitude of the winter epidemic peak suitable to explore the vaccine effectiveness in various epidemic conditions observed throughout the U.S. Our analysis highlights the importance of rolling out the vaccine fast and early which is highly relevant to the current epidemic situation given the spike in the confirmed cases locally and nationwide.

## Methods

### Model description

**Figure 1:**
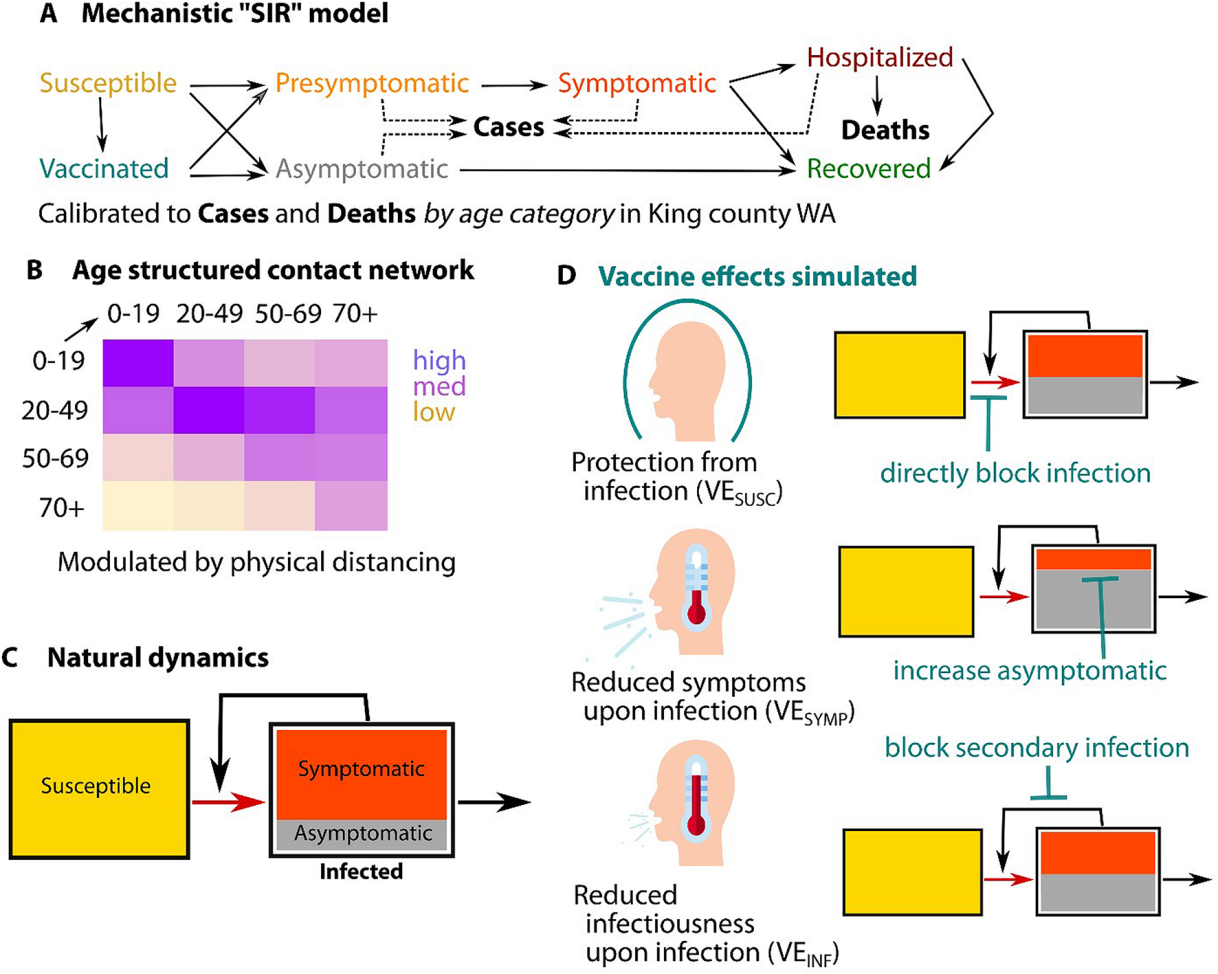
Schematic of the modeling analysis. (**A**) Diagram of the population transmission model with solid lines representing the flows of individuals between compartments and dotted lines indicating compartments which contribute to diagnosed cases; (**B**) mixing matrix between age groups showing proportions of contacts each group (row) has with the other groups (columns); (**C**) simplified diagram of the SARS-CoV2 transmission (red arrow) and the resulting force of infection (black arrow) in absence of vaccine and (**D**) the potential vaccine effects on the transmission and the force of infection.

We modified a previously developed deterministic compartment model which describes the epidemic dynamics in King County, WA^26^ to allow for vaccine rollouts. Our model (Fig. 1, Fig S1) stratifies the population by age (0–19 years, 20–49 years, 50–69 years, and 70+ years), infection status (susceptible, exposed, asymptomatic, pre-symptomatic, symptomatic, recovered), clinical status (undiagnosed, diagnosed, hospitalized) and vaccination status.

In our main scenario we assume that 20% of infections are asymptomatic reflecting the estimated proportion of SARS-CoV2 infections without symptoms in a published meta-analysis based on 79 studies^14^. We also assume that asymptomatic people are as infectious as symptomatic individuals but missing the highly infectious pre-symptomatic phase. As a result, the relative infectiousness of individuals who never express symptoms is 56% of the overall infectiousness of individuals who develop symptomatic COVID-19 infection. This estimate falls between the 35% relative infectiousness estimated in the meta-analysis^14^ and the current best estimate of 75% suggested by the CDC in their COVID-19 pandemic planning scenarios^27^. We explore alternative scenarios in which asymptomatic infections are only 28% less infectious than symptomatic cases to assess the importance of this assumption to the presented results.

The model is parameterized with local demographic and contact data from King County, WA and calibrated to local case and mortality data (Figs. S2, S3) using transmission parameter ranges informed from published sources (Tables S1, S2, S3)^28–31^. We used a genetic algorithm (NSGA-II multivariate optimization algorithm in the mco R package) and Monte Carlo filtering to select 100 calibrated parameter sets on epidemic start, transmission and hospitalization rates by age and physical isolation as a function of symptoms and diagnosis which reproduce the data from the Spring 2020 lockdown within pre-specified tolerances. We validated our assumptions on the diagnostic rates of symptomatic and asymptomatic cases during “reopening” period against independent data not used for calibration (cases and deaths after the calibration period) and independent region-specific modeling projections^32,33^. As a result, we obtained an ensemble of plausible epidemic trajectories with variable projections of the timing and magnitude of the winter epidemic peak suitable to explore the uncertainty in the vaccine effectiveness predictions associated with background epidemic conditions. We also explored additional scenarios in which only symptomatic cases get tested and diagnosed to assess the importance of this assumption for the projected impact of symptom averting vaccines. A full description of the model can be found in the Supplement.

### Vaccination scenarios

We consider four vaccine efficacy profiles (Table 1) with respect to vaccine effects on susceptibility (VE_SUSC_) and symptomatology (VE_SYMP_) which in combination determine the expected reduction in the likelihood to develop symptomatic diseases upon exposure (VE_DIS_) as described in Table 1. We compare two profiles (Vaccine 1 and Vaccine 2) satisfying the WHO and FDA requirement for COVID-19 vaccines to be at least 50% efficacious reducing the risk of symptomatic disease (50% VE_DIS_)^34,35^, and two profiles (Vaccine 3 and Vaccine 4) with efficacy against COVID-19 disease (90% VE_DIS_) comparable to the results reported on the Pfizer and Moderna vaccines^4,5^ and no effects on the infectiousness (VE_INF_ = 0) for either asymptomatic or symptomatic infections. The values explored for VE_SUSC_ and VE_SYMP_ allow us to clearly differentiate “susceptibility reducing” vaccines which actively reduce the risk to acquire infection (Vaccine 2 and 4) from “symptom reducing” vaccines which mostly convert symptomatic infections to asymptomatic (Vaccine 1 and 3).

**Table 1:** Vaccine efficacy profiles simulated in the main analysis.

Vaccine efficacy profiles	Vaccine 1	Vaccine 2	Vaccine 3	Vaccine 4
Vaccine effects on susceptibility (VE_SUSC_) defined as reduction of the probability to acquire infection upon exposure	0.1	0.4	0.1	0.8
Vaccine effects on symptomatology (VE_SYMP_) defined as reduction in the probability to develop symptoms upon infection	0.444	0.167	0.889	0.5
Vaccine effects on COVID-19 disease (VE_DIS_) = 1 − (1 − VE_SUSC_) (1 − VE_SYMP_) defined as reduction in the likelihood to develop symptomatic diseases upon exposure	0.5	0.5	0.9	0.9

All profiles assume no reduction in infectiousness (VE_INF_ = 0).

In our main scenario we assume 5000 vaccinations a day (~ 0.2% of the population) starting Dec 1, 2020 until 1,000,000 individuals are vaccinated, enough to cover ~ 45% of the population. The number of daily vaccinations gradually decreases when 90% of the coverage target is reached (see Fig S4). Daily vaccinations are distributed proportionally between susceptible and recovered classes which implies that individuals from those two groups are equally likely to be vaccinated. We do not explicitly model two-dose vaccine regimens but assume immediate immunization at the time when the vaccine regimen is completed. Our vaccination rate is comparable to the vaccination plan revealed by Biden administration^36^ assuming that vaccine doses will be distributed proportionally across regions but less optimistic compared to the prognosis by the Head of Operation Warp Speed who suggested that 70% of the U.S. population could be vaccinated by May^37^. Alternative scenarios with delayed vaccination start dates and 200,000–2,000,000 total vaccinated (10–90% population coverage) are also explored. Vaccinations are proportionally distributed across age groups without excluding individuals with prior SARS-CoV-2 infection.

### Metrics of interest

Population effectiveness is estimated as: (1) proportion of cumulative infections prevented; (2) proportion of cumulative hospitalizations prevented and (3) proportion of cumulative deaths prevented over 1 year after the vaccination start date compared to base-case scenarios in which vaccine is not available. Reductions in maximum daily infections, hospitalizations and deaths over 1 year from the vaccination start date are also evaluated. The uncertainty in the estimates is presented as 80% uncertainty interval (UI) based on simulations with 100 calibrated parameter sets per scenario.

## Results

### Base-case epidemic conditions

**Figure 2:**
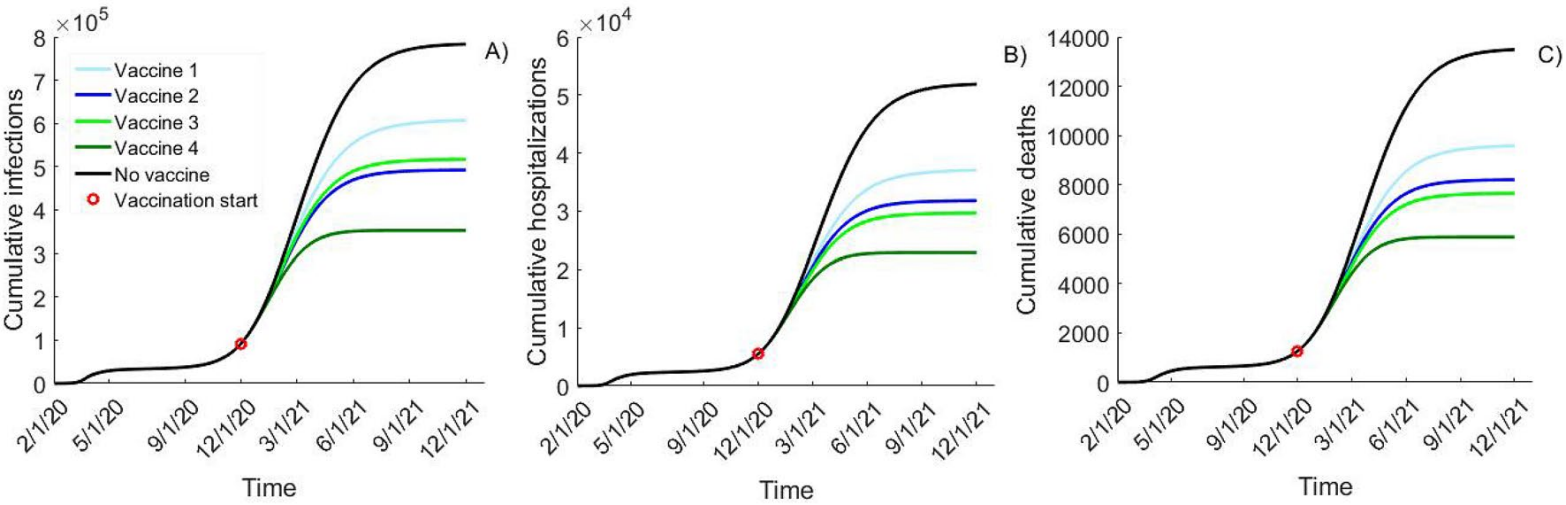
Epidemic projections with different vaccine efficacy profiles. Comparison of (**A**) cumulative infections; (**B**) cumulative hospitalizations and (**C**) cumulative deaths over time simulated with different efficacy profiles assuming vaccination start date of Dec. 1, 2020 and rolled out with 5000 vaccinated daily until 1,000,000 vaccinations are reached. Vaccine 1 (10% VE_SUSC_ and 44.4% VE_SYMP_) and Vaccine 2 (40% VE_SUSC_ and 16.7% VE_SYMP_) result in 50% reduction in symptomatic disease (VE_DIS_) while Vaccine 3 (10% VE_SUSC_ and 88.9% VE_SYMP_) and Vaccine 4 (80% VE_SUSC_ and 50% VE_SYMP_) result in 90% VE_DIS_. All projections represent the mean value of epidemic simulations using 100 calibrated parameter sets.

In the absence of a vaccine all calibrated simulations predict epidemic outbreaks reaching a maximum of 5900 (80% UI: 3700–8300) new infections, 390 (80% UI: 230–550) new hospitalizations and 99 (80% UI: 65–138) deaths daily at their transmission peak expected between January and July 2021. As of Dec. 1, 2020, the model projects that 4.2% of the population in King County is either infected or recovered with estimates across simulated epidemic conditions spanning over a range (80% UI: 1.4–8.5%) consistent with the COVID-19 seroprevalence projections for the majority of the US states^38^. The model projects 785,000 (80% UI: 647,000–914,000) cumulative infections and 13,500 (80% UI: 10,900–16,200) cumulative deaths by December 2021 if no vaccine becomes available (Fig. 2, black).

### Population impact of vaccines reducing the risk of COVID-19 disease by 50%

A “symptom reducing” vaccine with 50% VE_DIS_ (Vaccine 1: moderate VE_SYMP_, low VE_SUSC_) is projected to prevent 25% of the infections and 32% of the deaths over 1-year period reducing the cumulative number of infections by 180,000 to approximately 605,000 and the cumulative number of deaths by almost 4000 to approximately 9600 (Fig. 2, Vaccine 1). Vaccine 1 is projected to reduce the maximum number of daily hospitalizations and deaths by approximately 25% (Fig S5).

In comparison, a “susceptibility reducing” vaccine (Vaccine 2: moderate VE_SUSC_, low VE_SYMP_) with the same efficacy against COVID-19 disease (50% VE_DIS_) is expected to prevent additional 115,000 infections (17% more) and 1400 deaths (11% more) over 1 year (Fig. 2, Vaccine 2). These differences correspond to 65% and 35% relative increase in the population impact over the projections utilizing the “symptom reducing” (Vaccine 1), respectively.

### Population impact of vaccines reducing the risk of COVID-19 disease by 90%

A “symptom reducing” vaccine with 90% VE_DIS_ (Vaccine 3: low VE_SUSC_, high VE_SYMP_) is projected to have a comparable population impact to the “susceptibility reducing” vaccine with 50% VE_DIS_ (Vaccine 2). Vaccine 3 is projected to have a slightly smaller effect on SARS-CoV2 transmission than Vaccine 2 (39% vs 42% reduction over 1 year) while reducing 2100 more hospitalizations and 550 more deaths than Vaccine 2 (Fig. 2, light green vs. dark blue).

Finally, a “susceptibility reducing” vaccine with 90% VE_DIS_ (Vaccine 4: high VE_SUSC_, moderate VE_SYMP_) is projected to be the most effective at population level preventing additional 165,000 infections, 6800 hospitalizations and 1800 deaths over Vaccine 3 and reducing the cumulative infections to approximately 350,000 and cumulative deaths to approximately 5900 (Fig. 2, Vaccine 4).

**Figure 3:**
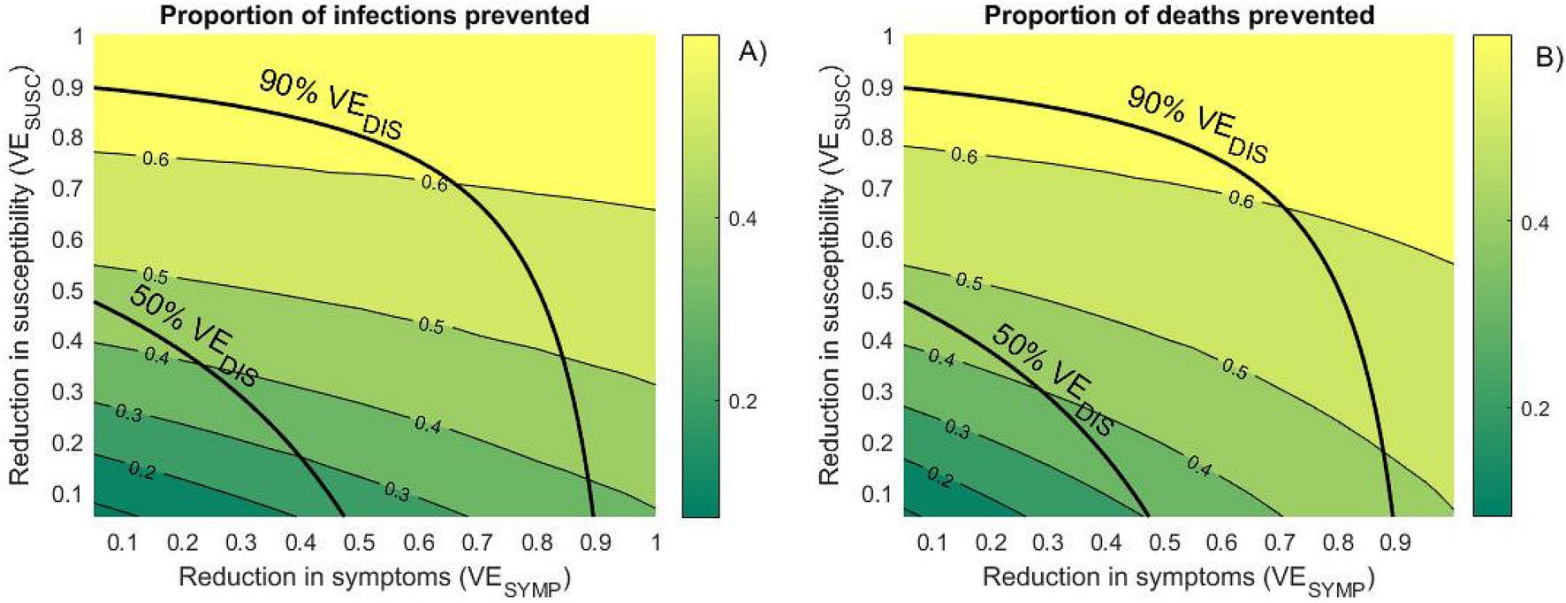
Projected vaccine effectiveness. Contour plots of the proportions of: (**A**) cumulative infections prevented and (**B**) cumulative deaths prevented over 1 year after the start of vaccine rollout by vaccines with different effects on susceptibility (VE_SUSC_) and the risk to develop symptoms (VE_SYMP_). Rollout assumes 5000 vaccinated daily till 1,000,000 vaccinations are reached. Thick lines represent VE profiles resulting in 50% and 90% reduction in symptomatic disease (VE_DIS_).

Figure 3 shows the wide range of population effectiveness which may result from the same reduction of the risk of COVID-19 disease observed in clinical studies. This uncertainty is particularly large for moderately effective vaccines (50% VE_DIS_) which may result in 23–46% reduction in transmission and 31–46% reduction in COVID-related mortality depending on which vaccine effects are induced. Vaccines with 90% VE_DIS_ are projected to prevent between 37 and 64% of the infections and between 46 and 64% of deaths over 1 year. Notably, the effectiveness ranges for moderate and highly effective vaccines overlap which suggest that “susceptibility reducing” vaccine with moderate efficacy against COVID disease may have greater population impact than highly effective “symptom reducing” vaccine. The importance of the effects on the disease severity (VE_SYMP_) decreases if the vaccine provides a strong protection against acquisition (VE_SUSC_) with the contour line corresponding to the same level of impact becoming more horizontal.

### It is critical to rollout the vaccine early and rapidly

Our analysis demonstrates that if the vaccine becomes available before the beginning of an outbreak it substantially increases the projected population impact (Fig S6). The 25 simulations with the earliest transmission peak (before February 10, 2021) average only 25% and 35% reduction in mortality for the vaccines with 90% VE_DIS_ (Vaccine 3 and Vaccine 4), respectively (Fig S6H,I, blue). In comparison, the same vaccines average 72% and 88% mortality reduction over the 25 simulations with the latest transmission peak after April 6, 2021 (Fig S6H,I, red).

**Figure 4:**
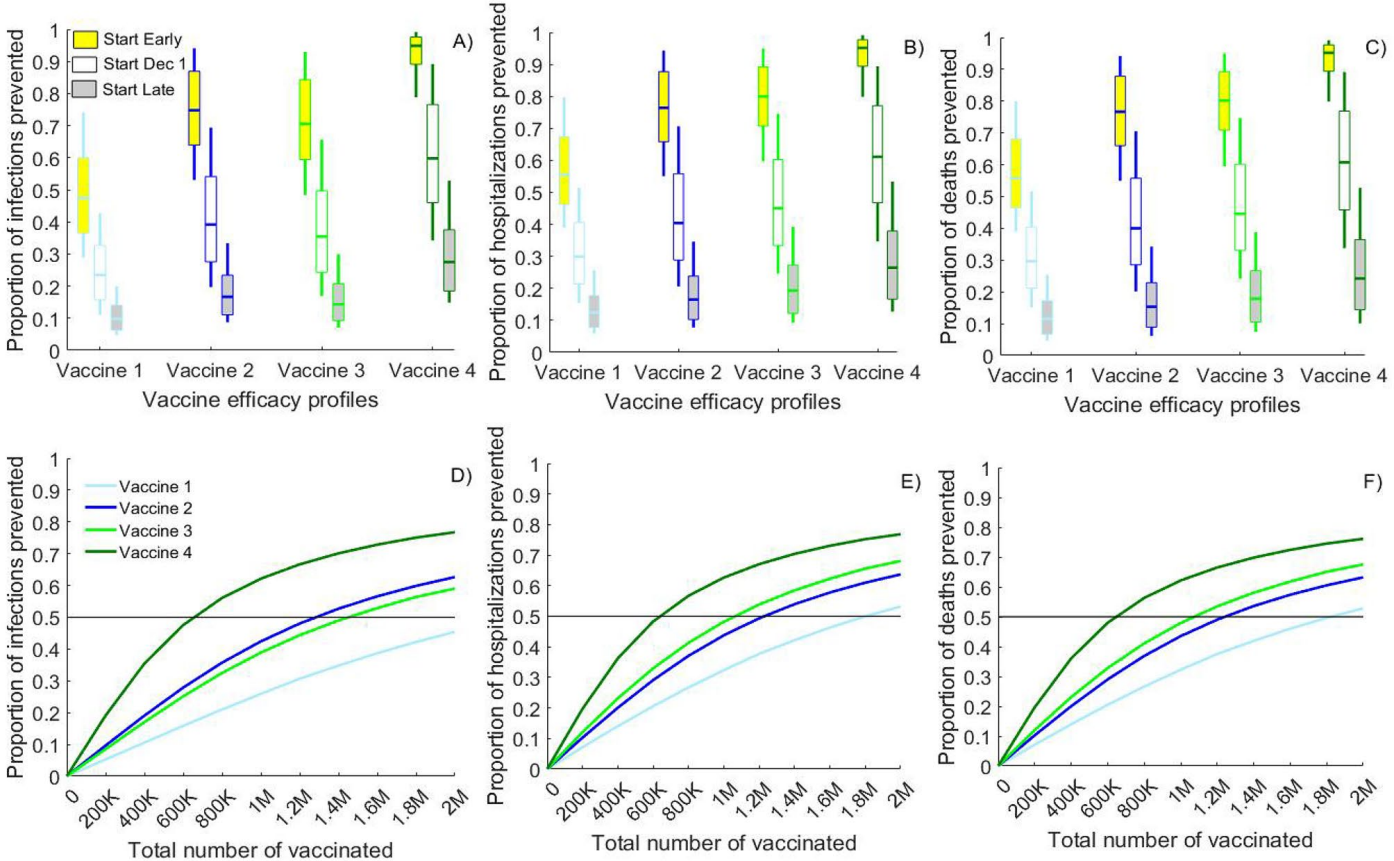
Importance of the timing and the coverage achieved with the vaccine rollout. Comparison of the projected reductions in the number of: (**A**,**D**) cumulative infections; (**B**,**E**) cumulative hospitalizations and (**C**,**F**) cumulative deaths over 1 year due to the use of vaccines with different efficacy profiles compared to base-case scenarios without vaccination. (**A**–**C**) compare vaccine rollouts which start on Dec. 1, 2020 (white boxes), Sept. 1, 2020 (yellow boxes) or March 1, 2021 (gray boxes) assuming 5000 vaccinated daily till 1,000,000 vaccinations are reached. (**D**–**F**) show the impact of rollouts starting on Dec. 1, 2020 and reaching different number of total vaccinations over 200 days. Vaccine 1 (10% VE_SUSC_ and 44.4% VE_SYMP_) and Vaccine 2 (40% VE_SUSC_ and 16.7% VE_SYMP_) result in 50% reduction in symptomatic disease (VE_DIS_) while Vaccine 3 (10% VE_SUSC_ and 88.9% VE_SYMP_) and Vaccine 4 (80% VE_SUSC_ and 50% VE_SYMP_) result in 90% VE_DIS_. Lines represent the mean value while boxplots represent the uncertainty generated by 100 calibrated simulations.

Delaying vaccination start to March 2021 decreases the expected population impact of the vaccination by approximately 50–56% in terms of transmission reduction, 52–56% in terms of hospitalization reduction and 55–58% in terms of mortality reduction compared to the main scenario assuming start at December 1, 2020. All simulated VE profiles are projected to prevent less than one third of the infections, hospitalizations and deaths over 1 year if the vaccination starts late (Fig. 4A–C, gray boxes). Conversely, if vaccines had been available even earlier (in Sept 2020) they would have averted additional 26–33% of the base-case infections, 25–32% of the base-case hospitalizations and 23–33% of the base-case deaths over 1 year (Fig. 4A–C, yellow boxes).

We also demonstrate the importance of fast vaccine rollout in response to concerns that only limited quantities will be available by Spring 2021. We project that only 650 K vaccinated (~ 30% coverage) with the “susceptibility reducing” Vaccine 4 (90% VE_DIS_, high VE_SUSC_) will be enough to reduce the COVID-19 transmission and mortality in half (Fig. 4D–F, dark green) in conjunction with physical distancing measures to keep physical interactions to 60% of pre-COVID levels (i.e., distancing, masking etc.). In comparison, the use of “symptom reducing” vaccine with the same 90% VE_DIS_ (Vaccine 3) will require 1.1 M vaccinations (~ 50% coverage) to achieve 50% reduction in mortality and 1.45 M vaccinations (~ 65% coverage) to achieve 50% reduction in transmission (Fig. 4D–F, light green). Averting three quarters of the annual transmission is impossible with Vaccine 1–3 even at 90% coverage but is achievable with 1.8 M vaccinated (~ 80% coverage) with Vaccine 4. Notably, at all coverage levels, the population impact of “susceptibility reducing” Vaccine 2 with moderate 50% VE_DIS_ is comparable with the impact of “symptom reducing” Vaccine 3 with high 90% VE_DIS_.

### When a vaccine rollout may result in small population impact?

In order to investigate the minimum impact that can be expected with a licensed vaccine with 50% or 90% VE_DIS_, we simulated a scenario in which: (1) no asymptomatic and pre-symptomatic cases are diagnosed, i.e., all SARS-CoV-2 testing is done after symptoms occur and (2) the overall infectiousness of individuals who never express symptoms is only 28% lower than symptomatic cases (Fig. S7). Under this pessimistic scenario “symptom reducing” vaccines are projected to reduce SARS-CoV-2 transmission by 5% (Vaccine 1) and 6% (Vaccine 3) and COVID-19 mortality by 9% (Vaccine 1) and 15% (Vaccine 3). These estimates should serve as a lower bound for the population effectiveness of licensed vaccines with unclear protection against infection. Notably, none of the vaccine profiles projected increased transmission for any of the simulated epidemic conditions.

## Discussion

The COVID-19 vaccines currently being tested must lower the number of symptomatic cases by 50% relative to placebo (50% VE_DIS_) in order to be licensed. Recent reports suggest that the first generation of mRNA vaccine have remarkable VE_DIS_ but their ability to prevent infection is currently not a criterion for approval and is unknown. Ultimately, seroconversion data from these studies may provide some insight regarding breakthrough asymptomatic infection but may be marked by loss of a humoral response such that underestimation of asymptomatic infection is possible.

In this analysis we explored different combinations of vaccine effects which result in 50% and 90% reduction of symptomatic COVID-19 and demonstrated significant variability in projected population effectiveness based on whether the vaccines fully prevent infection or convert symptomatic cases to asymptomatic cases with some ongoing possibility of transmission. Although this difference is not being evaluated as a primary endpoint in current studies, estimating the frequency and downstream transmission likelihood of asymptomatic infection is recognized as a research priority^11,39,40^. We identify that a high rate of conversion to asymptomatic infection is unlikely to increase the incidence of new cases. We demonstrate that the population effectiveness of “susceptibility reducing” vaccines can be up to 65% larger than “symptom reducing” vaccines even if resulting in the same observed VE_DIS_. Such vaccines contribute to a more rapid attainment of necessary vaccination thresholds to achieve herd immunity. Moreover, we found that a “susceptibility reducing” vaccine with moderate 50% VE_DIS_ may provide comparable population benefits with a “symptom reducing” vaccine with high 90% VE_DIS_. With multiple vaccine candidates in the pipeline, our results reveal the complexity of the problem and suggest that the usefulness of a vaccine should not be judged by a single efficacy number.

A significant proportion of the population impact expected with a “symptom reducing” vaccine can be attributed to the reduced infectiousness of the asymptomatic cases as has been reported for influenza vaccine^41^. This is despite the fact that asymptomatically infected people may remain infectious for a longer period of time given the lower likelihood of being diagnosed and self-isolating. Our analysis suggests that the population effectiveness of “symptom reducing” vaccine, measured as reduction in SARS-CoV2 transmission, may decrease to below 10% if the infectiousness gap between asymptomatic and symptomatic cases is smaller and if individuals who do not show symptoms are not readily tested, diagnosed and isolated. This highlights the need to continue the contact tracing and quarantine measures, currently in place, for at least the duration of the vaccine rollout.

We also conclude that vaccine rollout will be significantly more effective if introduced prior to rather than during a surge in cases. Even the rollout of a vaccine with 90% VE_DIS_ may result in very small reduction in actual SARS-CoV-2 transmission if it occurs after a substantial number of people have been already infected. Unfortunately, at the time of this writing, the effective reproductive number exceeds one in virtually every U.S. state and many hospitals are already nearing capacity in many regions of the country. During the current surge, there is little evidence as of yet that case incidence has started to lower as states in the Northern Plains now have an estimated prevalence approaching 25% and few US states have reimposed lockdowns^42,43^. On November 15, 2020 Washington state (and thus King County, the focus of this paper) reimposed some restrictions closing businesses and limiting gatherings but the impact of these measures has not affected the upward epidemic trajectories yet^44^. If the current uncontrolled increase in confirmed cases and hospitalizations across the U.S. continues, the expected vaccination in Spring 2021 may come too late. The most recent projections of the Institute for Health Metrics and Evaluations predict that the trend of increasing cases in Washington State will continue until February 2021^43^. Our analysis provides a strong rationale for mitigation measures such as mask mandates^45^ and further physical distancing^46,47^ to contain the epidemic surge and weather the incoming months in expectation of mass production and distribution of safe and effective vaccines.

Our results qualitatively agree with previous studies^17,23^ that supporting non-pharmaceutical measures might be needed during vaccination campaigns to slow down the epidemic and increase its effectiveness. We also share the conclusion of other modeling teams that vaccines mediated by a reduction in susceptibility to infection will have a greater impact than disease-modifying vaccines^24,25^. However, our analysis suggests that mortality reduction greater than 75%, projected in some studies^23^, is achievable under high vaccine coverage (50%) only if a “susceptibility reducing” vaccine is rolled out rapidly in well-controlled epidemic environment but not in the middle of an epidemic wave. The importance of an early vaccine rollout has also been highlighted in other studies^19^.

Some caveats of this work are important to note. First, we assumed that the licensed vaccines have no direct effect on the infectiousness (VE_INF_) of the breakthrough infections. Nevertheless, the vaccine shifting symptomatic infections to less infectious asymptomatic infection indirectly reduces overall infectiousness. While a moderate VE_INF_ will not directly protect the vaccine recipient, it would likely effectively limit further chains of transmission and therefore should be evaluated. Second, we do not explicitly model two-dose vaccine regimens, yet our results point to early and rapid rollouts as a key for a successful vaccination campaign. We expect that employing more realistic dose distribution (explicitly modeling two doses and lead-time to mount protective immunity) will strengthen these conclusions. Third, we considered vaccination rollouts where all age groups are vaccinated. Current recommendations point to vaccinating older age groups first, which are those at higher risk. In that sense, our model might be underestimating the impact of vaccination rollouts on the number of deaths prevented. We also exclude from vaccination individuals who are currently infected with SARS-CoV-2. Even if some asymptomatic infected may get vaccinated they will be very small number compared to those from susceptible and recovered groups. This will result in slightly more vaccine doses being “wasted” in addition to all recovered individuals who become vaccinated while being fully protected. Forth, we assume that the level of physical interactions remains steady for the next year which results in epidemic outbreaks of various magnitudes providing a reasonable variability in the counterfactual epidemic conditions in which we evaluate vaccines with different efficacy profiles. Enforcing partial or full lockdowns and reopenings would likely result in periodic fluctuations in the number of infections and confirmed cases^48^ but unlikely to affect effectiveness projections if the same schedule is applied to scenarios with and without vaccination. However, if physical interactions increase with the rollout of vaccines either due to policy shifts or individual behavioral compensation, the resulting outcomes would be worse than what we have predicted here. Finally, the emergence and spread of more contagious variants may change the epidemic dynamics in King County but unlikely to affect the population impact differences between “susceptibility reducing” and “symptom reducing” vaccines, reported here, provided that the vaccines are equally effective against currently prevalent and new strains. Careful monitoring of the spread of new variants and further research on these questions is warranted.

The results of this analysis suggest that fully understanding the efficacy profile of the vaccine is important, since vaccines with different profiles may show similar efficacy in clinical studies but have considerably different population impact. In particular, vaccines which prevent COVID-19 disease but not SARS-CoV-2 infection, and thereby shift symptomatic infections to asymptomatic infections, will prevent fewer infections and require larger and faster vaccination rollouts to achieve the same population impact as vaccines that reduce susceptibility to infection.

## Supplementary Information

Supplementary Information 1.
