# Supplementary material for: COVID-19 vaccines that reduce symptoms but do not block infection need higher coverage and faster rollout to achieve population impact
Source: Sci Rep. 2021 Jul 30;11:15531. doi: 10.1038/s41598-021-94719-y (PMC8324774; doi:10.1038/s41598-021-94719-y)
Supplement: Supplementary file 1 — Supplementary Information 1. [file 41598_2021_94719_MOESM1_ESM.docx]

**Supplementary Information to**

**COVID-19 vaccines that reduce symptoms but do not block infection need higher coverage and faster rollout to achieve population impact**

David A. Swan^1^†, Chloe Bracis^2^†, Holly Janes^1^, Mia Moore^1^, Laura Matrajt^1^, Daniel B. Reeves^1^, Eileen Burns^3^, Deborah Donnell^1,4^, Myron S. Cohen^5^, Joshua T. Schiffer^1,6,7^††, Dobromir Dimitrov^1,8*^††

^1^Vaccine and Infectious Disease Division, Fred Hutchinson Cancer Research Center, Seattle, WA, USA

^2^Université Grenoble Alpes, TIMC-IMAG / BCM, 38000, Grenoble, France

^3^Independent Researcher, Seattle, WA, USA

^4^Department of Global Health, University of Washington, Seattle, WA USA

^5^Department of Epidemiology, University of North Carolina at Chapel Hill, Chapel Hill, NC USA

^6^Clinical Research Division, Fred Hutchinson Cancer Research Center; Seattle, WA, USA

^7^Department of Medicine, University of Washington, Seattle, WA, USA

^8^Department of Applied Mathematics, University of Washington, Seattle, WA, USA

† These authors contributed equally to the work.

†† These authors contributed equally to the work.

*Corresponding author email: [ddimitro@fredhutch.org](mailto:ddimitro@fredhutch.org)

**1. Complete model description**

**
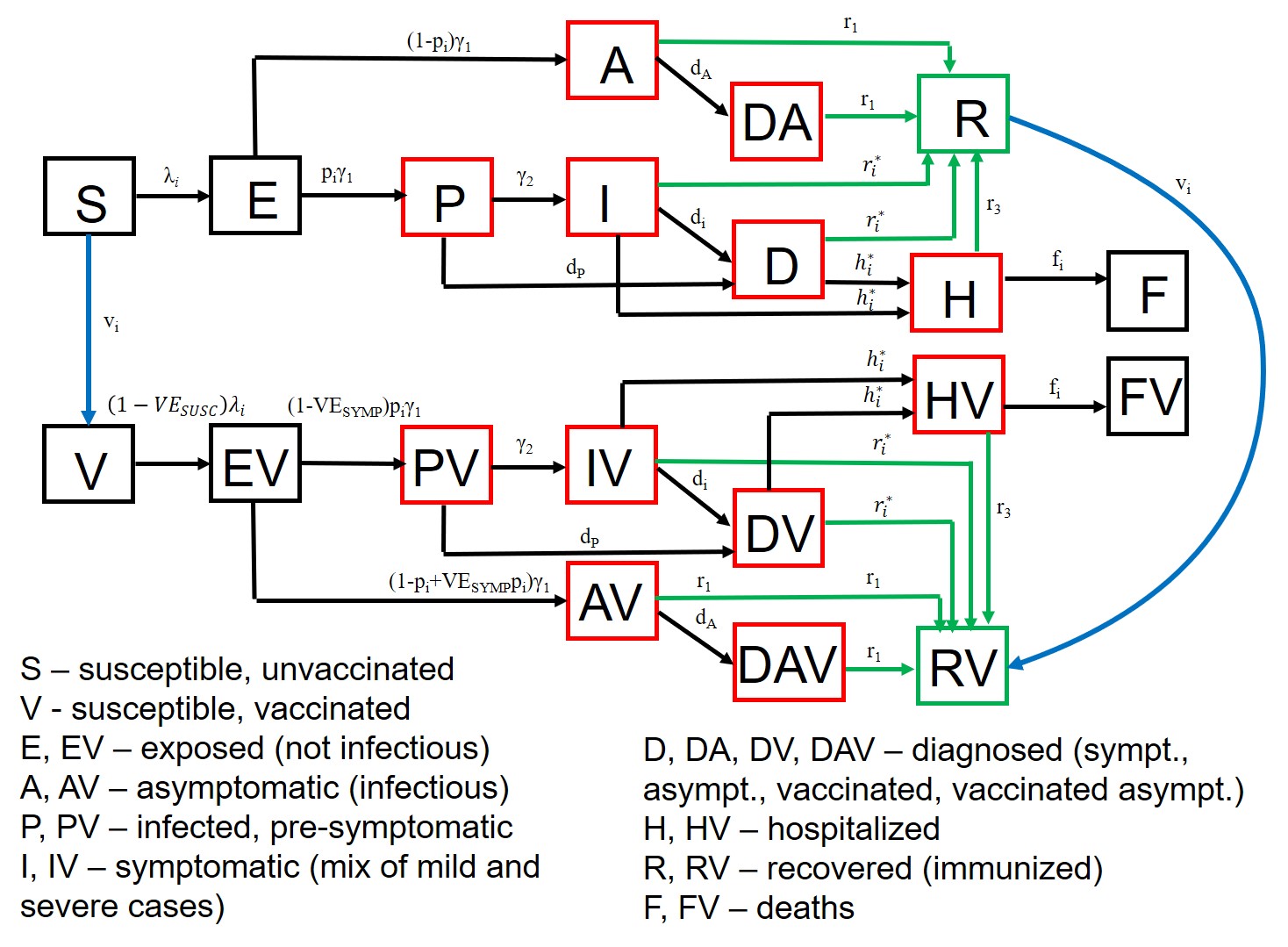
**

**Figure S1. Model diagram** Structure of the mathematical model of SARS-CoV-2 dynamics. Stratification by age (4 groups) is not shown. Red compartments are infectious while green compartments are recovered with long-lasting natural immunity. Blue arrows indicate vaccination.

**The model is described by a set of differential equations for each age group (i=1 for** **age 0-19 years, i=2 for age 20-49 years, i=3 for age 50-69 years, and i=4 for age 70+ years):**

$$\frac{dS_{i}}{dt}=-\lambda_{i}S_{i}-\frac{{v_{i}S}_{i}}{S_{i}+R_{i}}$$

$$\frac{dE_{i}}{dt}=\lambda_{i}S_{i}-\gamma_{1}E_{i}$$

$$\frac{dA_{i}}{dt}=\left( 1-p_{i} \right)\gamma_{1}E_{i}-{(r}_{1}+d_{A})A_{i}$$

$$\frac{d{DA}_{i}}{dt}=d_{A}A_{i}-r_{1}{DA}_{i}$$

$$\frac{dP_{i}}{dt}=p_{i}\gamma_{1}E_{i}-{(\gamma}_{2}+d_{P}{)P}_{i}$$

$$\frac{dI_{i}}{dt}=\gamma_{2}P_{i}-d_{i}{\left( t \right)I}_{i}-{h_{i}^{*}I}_{i}-r_{i}^{*}I_{i}$$

$$\frac{dD_{i}}{dt}={d_{P}P_{i}+d}_{i}(t)I_{i}-h_{i}^{*}D_{i}-r_{i}^{*}D_{i}$$

$$\frac{dH_{i}}{dt}=h_{i}^{*}I_{i}+h_{i}^{*}D_{i}-r_{3}H_{i}-f_{i}H_{i}$$

$$\frac{dF_{i}}{dt}=f_{i}H_{i}$$

$$\frac{dR_{i}}{dt}=r_{1}A_{i}+{r_{1}{DA}_{i}+r_{i}^{*}}I_{i}+r_{i}^{*}D_{i}+r_{3}H_{i}-\frac{{v_{i}R}_{i}}{S_{i}+R_{i}}$$

$$\frac{dV_{i}}{dt}=-\left( 1-{VE}_{SUSC} \right)\lambda_{i}V_{i}+\frac{{v_{i}S}_{i}}{S_{i}+R_{i}}$$

$$\frac{d{EV}_{i}}{dt}=\left( 1-{VE}_{SUSC} \right)\lambda_{i}V_{i}-\gamma_{1}{EV}_{i}$$

$$\frac{d{AV}_{i}}{dt}=\left( 1-p_{i}+{{VE}_{SYMP}p}_{i} \right)\gamma_{1}{EV}_{i}-{(r}_{1}+d_{A}){AV}_{i}$$

$$\frac{d{DAV}_{i}}{dt}=d_{A}{AV}_{i}-r_{1}{DAV}_{i}$$

$$\frac{d{PV}_{i}}{dt}={(1-{VE}_{SYMP})p}_{i}\gamma_{1}{EV}_{i}-{(\gamma}_{2}+d_{P}{)PV}_{i}$$

$$\frac{d{IV}_{i}}{dt}=\gamma_{2}{PV}_{i}-d_{i}{\left( t \right)IV}_{i}-{h_{i}^{*}IV}_{i}-r_{i}^{*}{IV}_{i}$$

$$\frac{d{DV}_{i}}{dt}={d_{P}{PV}_{i}+d}_{i}(t){IV}_{i}-h_{i}^{*}{DV}_{i}-r_{i}^{*}{DV}_{i}$$

$$\frac{d{HV}_{i}}{dt}={h_{i}^{*}IV}_{i}+h_{i}^{*}{DV}_{i}-r_{3}{HV}_{i}-f_{i}{HV}_{i}$$

$$\frac{d{FV}_{i}}{dt}=f_{i}{HV}_{i}$$

$$\frac{d{RV}_{i}}{dt}=r_{1}{AV}_{i}+{r_{1}{DAV}_{i}+r_{i}^{*}}{IV}_{i}+r_{i}^{*}{DV}_{i}+r_{3}{HV}_{i}+\frac{{v_{i}R}_{i}}{S_{i}+R_{i}}$$

*p_i_* – proportion of the infections which become symptomatic by age in absence of a vaccine

γ*_1,_* γ*_2_*- progression rates from exposed (E) to infectious (A and P) to symptomatic (I)

$h_{i}^{*}$ – hospitalization rate among all symptomatic cases (diagnosed or not) by age (calculated, see Table S1)

*r_1,3_ -* recovery rate of the asymptomatic and hospitalized cases

$r_{i}^{*}$ *-* recovery rate among all symptomatic cases (diagnosed or not) by age (calculated, see Table S1)

$f_{i}$ – fatality rate among hospitalized by age

${VE}_{SUSC}$ – vaccine efficacy in reducing susceptibility to infection

${VE}_{SYMP}$ – vaccine efficacy in reducing the risk of symptomatic disease after infection

$v_{i}$ – daily vaccinations by age. They are based on fixed daily number of vaccinations distributed proportionally across age groups ($\bar{v}_{i}$) which gradually decreases after 90% of the overall vaccination target (*V_tot_*) is reached as follows: $v_{i}=\left\{ \begin{aligned} \bar{v}_{i}, cum.vacc<{0.9 V}_{tot} \\ {0.8 \bar{v}}_{i}, {0.9 V}_{tot}<cum.vacc<{0.925 V}_{tot} \\ {0.6 \bar{v}}_{i}, {0.925 V}_{tot}<cum.vacc<{0.95 V}_{tot} \\ {0.4 \bar{v}}_{i}, {0.95 V}_{tot}<cum.vacc<{0.975 V}_{tot} \\ {0.2 \bar{v}}_{i}, {0.975 V}_{tot}<cum.vacc \end{aligned} \right.$

*d_i_* – diagnostic rate by age. They vary in time being initially set at zero and later elevated after the start of the COVID measures at t=δ_1_. Finally, they are further elevated after testing capacity was increased t=δ_3_, i.e. $d_{i}\left( t \right)=\left\{ \begin{aligned} 0, t<\delta_{1} \\ d_{i,1}, \delta_{1}\leq t< \delta_{3} \\ d_{i,2}, t\geq\delta_{3} \end{aligned} \right.$

The forces of infection ($\lambda_{i})$, representing the risk of the susceptible individuals by age to acquire infection (transition from susceptible to exposed), are differentiated by age of the susceptible individual, the contact matrix (proportion of contacts with each age group), infection and treatment status (asymptomatic, pre-symptomatic, symptomatic, diagnosed and hospitalized cases) of the infected contacts, and the time-dependent reduction of transmission due to physical distancing measures (work from home, closing non-essential businesses, banning large gathering, etc,) applied in the area (scaled up starting March 8 and fully taking effect March 29) and later relaxed during the reopening after May 15.

$$\lambda_{i}=\sum_{j=1}^{4} c_{ij} \left( {1-R_{sd}^{j}}\left( t \right) \right)\left[ \beta_{a}A_{j}+\beta_{p}P_{j}+\beta_{s}I_{j}+\beta_{d}D_{j}+ \beta_{da}{DA}_{j}+\left( 1-{VE}_{INF} \right)\left( \beta_{a}{AV}_{j}+\beta_{p}{PV}_{j}+\beta_{s}{IV}_{j}+\beta_{d}{DV}_{j}+ \beta_{da}{DAV}_{j} \right) \right]/N_{j}+{c_{ij}\beta}_{h}(H_{j}+{HV}_{j})/N_{j}$$

where

*β_a_*, *β_p_*, *β_s_*, *β_d_*, *β_da_*, *β_h_* are the transmission rates from contacts with asymptomatic, pre-symptomatic, symptomatic, diagnosed symptomatic, diagnosed asymptomatic, and hospitalized infections (before the start of COVID mitigation measures at t= δ_1_),

*c_ij_* – contact matrix (proportion of contacts between age groups),

*N_i_* – population size by age,

${VE}_{INF}$ – vaccine efficacy in reducing the infectiousness

$R_{sd}^{i}$ (t) is the reduction in SARS-CoV2 transmission due to distancing, masking and other preventive measures compared to pre-COVID levels which is initially applied uniformly to all age groups. It is scaled up linearly from 0 to $R_{sd}^{max}$between t= δ_1_ and t= δ_2_). Later it is decreased to 40% for all age groups except seniors during reopening period (between t= δ_3_ and t= δ_4_).

**2. Model parameterization**

In our main scenario we assume that 20% of infections are asymptomatic reflecting the estimated proportion of SARS-CoV2 infections without symptoms in a published meta-analysis based on 79 studies.^1^ We also assume that asymptomatic people are as infectious as symptomatic individuals but missing the highly infectious pre-symptomatic phase. As a result, the relative infectiousness of individuals who never express symptoms is 56% of the overall infectiousness of individuals who develop symptomatic COVID-19 infection. This estimate falls between the 35% relative infectiousness estimated in the meta-analysis^1^ and the current best estimate of 75% suggested by the CDC in their COVID-19 pandemic planning scenarios^2^. We explore alternative scenarios in which the difference in overall infectiousness between asymptomatic and symptomatic cases is smaller (asymptomatic infections are only 28% less infectious than symptomatic cases) to assess the importance of this assumption to the presented results.

**Table S1. Parameters and ranges used in the analysis. (Fixed in black, Scenarios in blue, Calibration in red)**

| **Parameter** | **Description** | **Values and ranges** | **Type** |
| --- | --- | --- | --- |
| ***γ_1_*** | **Progression rates from exposed (E) to infectious (A or P) (latent time)^-1^** | **(3 days)^-1^** | Fixed |
| ***γ_2_*** | **Progression rates from pre-symptomatic (P) to symptomatic (I) (pre-symptomatic time)^-1^** | **(2 days)^-1^** | Fixed |
| ***p_i_*** | **Proportion of the infections which become symptomatic by age** | **80%** | **Fixed** |
| ***v_i_*** | **Vaccination rate by age. Number of currently uninfected individuals who get vaccinated daily for 200 days after the start of vaccination** | Proportional by age-group size (see Table S3)  Main: 5000  Alternative: 1000-10000 | **Scenarios** |
| $d_{i,j}$ | **Diagnostic rate if symptomatic by age (*i*) after COVID measures are initiated (*j=1*) and after testing capacity was increased (*j=2*)** | ***d_i,1_*=1-10%**  ***d_i,2_*=10%** | Calibrated |
| $d_{A},d_{P}$ | **Diagnostic rate if asymptomatic and pre-symptomatic before (*j=1*) and after (*j=2*) testing capacity was increased** | ***d_A,1_*= *d_P,1_*=0**  ***Main: d_A,2_*= *d_P,2_*=5%**  ***Alternative: d_A,2_*= *d_P,2_*=0** | Scenarios |
| ***id*** | **Symptomatic infectiousness duration** | **7 days** | Fixed |
| *β_i_* | **Daily transmission from infected from asymptomatic, pre-symptomatic, symptomatic, diagnosed asymptomatic, diagnosed symptomatic and hospitalized groups in absence of COVID measures** | ***=βs*(1, β_p_, 1, β_d_, β_d_, 0)***  ***β_p_*** calculated to get 44% pre-sympt. transmission  ***β_s_ =R0/(βp/γ_2_ + id) for R0 = 2.2-4***  ***β_d_ =0.5-0.75 (Lockdown)***  ***Decreased 50% afterward*** | Calculated  Calibrated |
| $R_{sd}^{max}$ | **Maximal reduction of transmission due to social distancing (scaled up linearly between t= δ_1_ and t= δ_2_)** | ***50%-90%*** | Calibrated |
| **δ_0_** | **Number of days between the start of the simulation (day 0) and the 1^st^ diagnosed case from data (Feb.28)** | ***40-50*** | Calibrated |
| **[δ1, δ2]** | **Period of scaling up COVID measures** | **March 8-29** | Fixed |
| **[δ_3_, δ_4_]** | **Reopening period** | **May 15- July 15** | Fixed |
| *m_i_* | **Proportion of symptomatic case which remain mild by age** | **99.8%, 96.9%, 87.1%, 74.5%** | Fixed |
| *h_i_* | **Hospitalization rate among severe cases by age** | ***h_1_=0.1, h_2_=0.15, h_3_=0.15-0.3, h_4_=0.15-0.3*** | Calibrated |
| $h_{i}^{*}$ | **Hospitalization rate among symptomatic cases (diagnosed or not)** | $=\left( \boldsymbol{1-}\boldsymbol{m}_{\boldsymbol{i}} \right)\boldsymbol{h}_{\boldsymbol{i}}$ | Calculated |
| ***r_1_*** | **Recovery rate of asymptomatic cases** | ***Main: 1/id,***  ***Alternative: 1/9*** | Scenarios |
| ***r_2_*** | **Recovery rate of mild symptomatic cases (who don’t need hospitalization)** | ***1/id*** | Fixed |
| $r_{i}^{*}$ | **Recovery rate of symptomatic cases** | $=m_{i}r_{2}$ | Calculated |
| ***r_3_*** | **Recovery rate of the hospitalized cases** | ***1/14*** | Fixed |
| ***hd*** | **Time from hospitalization to death** | **11.2 days** | fixed |
| $f_{i}$ | **Fatality rate by age among hospitalized before reaching ICU capacity* (overall mortality when hospitalized/time to death) adjusted for underreporting of mild cases** | **=α*CFR/(1-m_i_)/hd**  **CFR: (0%,0.2%,2.1%,15.9%)**  **α =0.8-1.25** | Calibrated |
| ***V_tot_*** | **Total number of vaccinations** | **Main: 1,000,000**  **Alternative: 200K-2M** | **Scenarios** |
|  | **Daily vaccinations** | **Main: 5000**  **Alternative: 1000-10,000** | **Scenarios** |

**Table S2. Contact matrix**. We used a previously published age-structured contact matrices created from population-based prospective survey of mixing patterns in different European countries.^3^ Our contact matrix is using specifically data from UK. The columns represent the distribution of contacts of a person from given age group across all age groups:

| Proportion contacts with | 0-19 y | 20-49 y | 50-69 y | 70+ y |
| --- | --- | --- | --- | --- |
| 0-19 y | 0.56 | 0.24 | 0.15 | 0.18 |
| 20-49 y | 0.34 | 0.57 | 0.49 | 0.34 |
| 50-69 y | 0.08 | 0.16 | 0.29 | 0.28 |
| 70+ y | 0.01 | 0.03 | 0.07 | 0.20 |

**Table S3. King County age pyramid based on data from 2017**

| Proportion of the population | 0-19 y | 20-49 y | 50-69 y | 70+ y |
| --- | --- | --- | --- | --- |
|  | 22.93% | 45.52% | 23.50% | 8.05% |

**3. Model Calibration**

The model is calibrated to 5 “targets” based on local data **(Fig S2)**, including: i) overall number of confirmed cases (target #1) and deaths (target #2) reported daily in King County over time since the start of the epidemic outbreak through April 30; ii) age-distribution of the cumulative confirmed cases (target #3) and deaths (target #4) reported daily in King County at four time points after the start of the epidemic outbreak (29 March, 7 April, 11 April, and 15 April 2020) and iii) the timing of the peak of daily confirmed cases (target #5) estimated as April 1. For each target, we calculated the mean squared error (MSE) between the target and the model output, summing the MSEs in the case where we had multiple values through time (all but target #5). We used a genetic algorithm (NSGA-II multivariate optimization algorithm in the mco R package) to evolve a population of parameterizations to arrive at a set approximating the Pareto front. From the Pareto front, we were interested in selecting the parameterization providing a good fit to all five criteria (versus some parameterizations that fit one criterion extremely well but others not at all). We defined the following thresholds for the MSE for each target:

- ***target #1: 2e7;***
- ***target #2: 5e4;***
- ***target #3: 1000;***
- ***target #4: 2000 and***
- ***target #5: 25***

which ensured a reasonable fit to these targets and were based on the errors associated to the best fit obtained with the genetic algorithm.

In order to characterize the uncertainty in the model, we generated 100 alternative parameterizations that also met these threshold criteria of providing a reasonable fit to the data using Monte Carlo filtering. We generated parameterizations by randomly sampling parameters within the ranges used for calibration (some ranges were narrowed based on the results of the calibration to make the algorithm more efficient) then used rejection sampling to retain just those sets that met all five threshold criteria and stopping when we had retained 100 parameter sets. As a result, we obtained an ensemble of 100 calibrated epidemic trajectories (see **Fig S2**) with variable projections of the timing and magnitude of the winter epidemic peak suitable to explore the uncertainty in the vaccine effectiveness predictions associated with background epidemic conditions. We also explored additional scenarios in which only symptomatic cases get tested and diagnosed to assess the importance of this assumption for the projected impact of symptom averting vaccines.


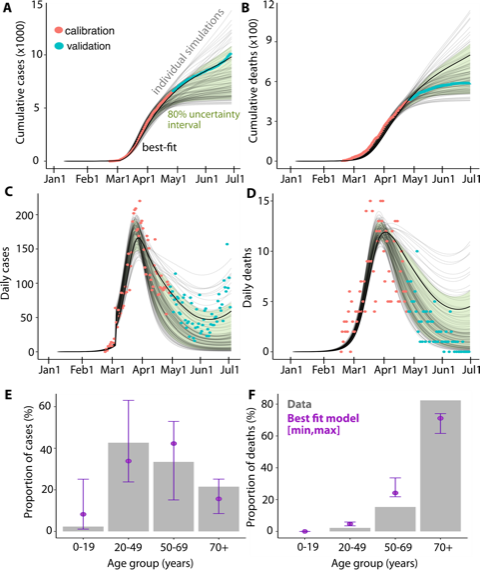


**Figure S2.** **Model calibration and validation.** Model fitting to 5 sources of King County data assuming gradual scale up of social distancing between March 8 and March 29: A)-D) Cumulative and daily cases and deaths. Red dots represent data up to April 30, thick lines represent the best model fit while other acceptable trajectories are shown in grey. Green bands show 80% range from acceptable trajectories. E)-F) Age distributions of cases and deaths as of April 15. Bars represent data, purple dots and ranges represent the best fit and other acceptable trajectories included in the analysis. Reopening plan is implemented between May 15 and July 15 by gradually restoring 60% of the physical interactions for all age groups (reducing *R_sd_* (t) to 40%) compared to pre-COVID levels.


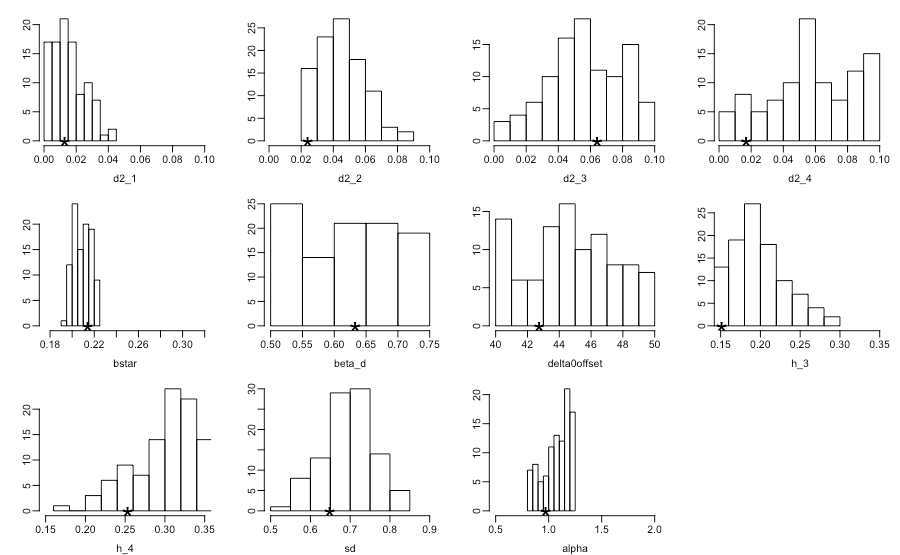


**Figure S3. Calibrated parameter sets**. Shown are the distributions of the parameter values for the 100 acceptable parameterizations (UI) with a * marking the value of the ‘best’ parameterization.

We validate our population model by predicting independent data not used for calibration of cumulative number of confirmed cases and deaths between April 30 and June 30, as well as estimated number of daily hospitalizations and the overall hospitalization rates among confirmed cases at the end of April. We also compared to independent region-specific modeling projections including cumulative SARS-CoV-2 incidence at the beginning of March based on genomic analyses^4^ as well as the cumulative incidence and effective reproductive number (Rt) estimates for King County for the period between March 1 and April 22 reported by the Institute for Disease Modeling^5^.

**4. Vaccination scenarios**

In our main scenario we assume 5000 vaccinations a day starting Dec 1, 2020 until 1,000,000 individuals are vaccinated, enough to cover ~45% of the population. The number of daily vaccinations gradually decreases when 90% of the coverage target is reached (see **Fig S4**). As a result, the last vaccination occurs 233 days after the vaccination start. We assume that fixed number of vaccinations (*v_i_*) are done daily in each age group. They are distributed proportionally between susceptible and recovered classes which implies that individuals from those 2 groups are equally likely to be vaccinated. As a result $\frac{\boldsymbol{v}_{\boldsymbol{i}}\boldsymbol{S}_{\boldsymbol{i}}}{\boldsymbol{S}_{\boldsymbol{i}}\boldsymbol{+}\boldsymbol{R}_{\boldsymbol{i}}}$and $\frac{\boldsymbol{v}_{\boldsymbol{i}}\boldsymbol{R}_{\boldsymbol{i}}}{\boldsymbol{S}_{\boldsymbol{i}}\boldsymbol{+}\boldsymbol{R}_{\boldsymbol{i}}}$ are vaccinated from each group. Our vaccination rate and population coverage are less optimistic compared to the recent prognosis by the Head of Operation Warp Speed who suggested that 70% of the U.S. population could be vaccinated by May.^6^ Alternative scenarios with delayed vaccination start dates and 200,000-2,000,000 total vaccinated (10%-90% population coverage) are also explored. Vaccinations are proportionally distributed across age groups without excluding individuals with prior SARS-CoV-2 infection. Vaccinated individuals are assumed to continue with the same level of physical interactions as unvaccinated, i.e. there is no behavioral disinhibition. We don’t explicitly model two-dose vaccine regimens but assume immediate immunization at the time of vaccination.


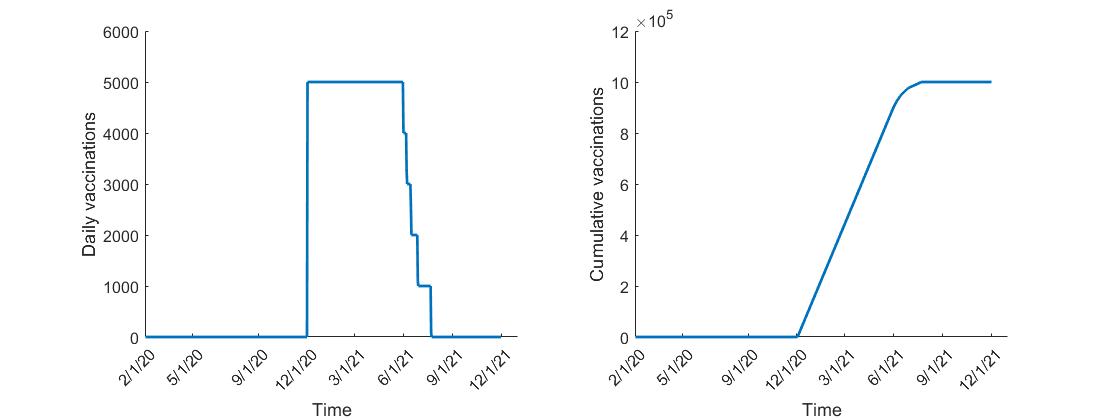


**Figure S4. Vaccination rollout in the main scenario.** A) Daily and B) Cumulative number of vaccinations done over time till the target of 1,000,000 vaccinations is reached.

**5. Additional results**


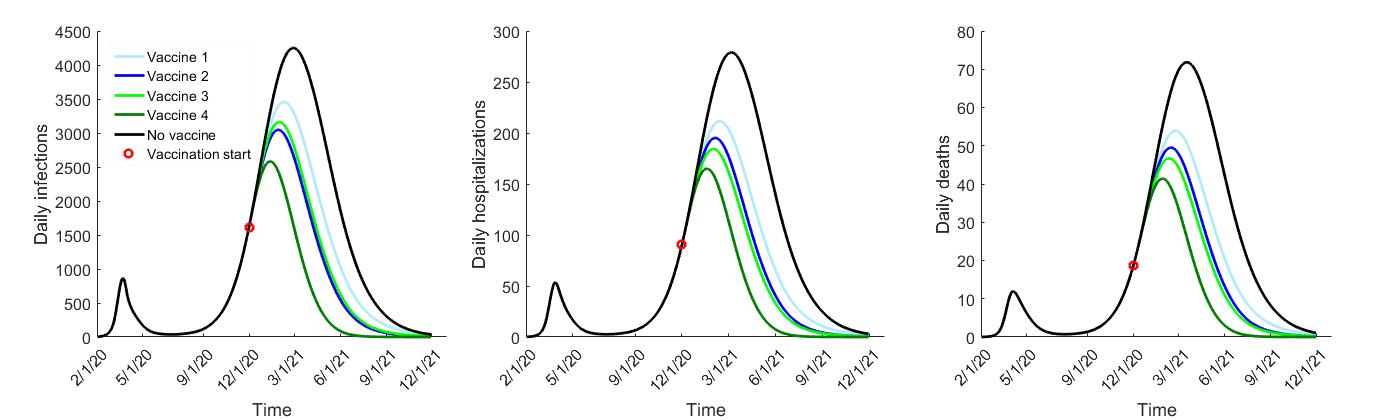


A)

B)

C)

**Figure S5.** **Average daily transmission, hospitalization and mortality curves based on simulations with calibrated parameter sets.** Vaccine 1 and 2 result in 50% reduction in symptomatic disease (VE_DIS_) while Vaccine 3 and 4 result in 90% VE_DIS_. Vaccine rollout begins on Dec.1 with 5,000 vaccinated daily till 1,000,000 vaccinations are reached.


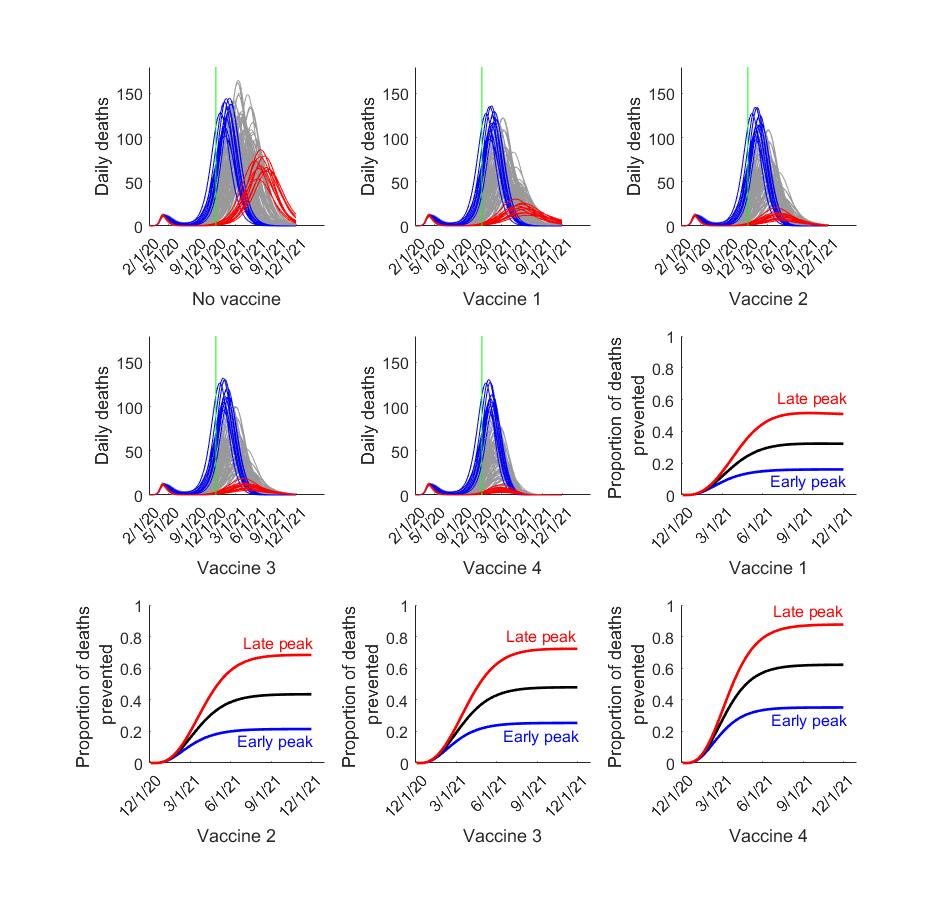


A)

B)

C)

D)

E)

F)

G)

H)

I)

**Figure S6** **Association between outbreak transmission peak and vaccine effectiveness** Daily mortality curves A) without vaccination and B)-E) with different vaccine profiles for all 100 calibrated simulations. Simulations with the 10 highest reductions of mortality are colored in red while the 10 lowest are colored in blue. Green vertical lines indicate vaccination start date. F)-I) Projected mortality reduction for different vaccine profiles when based on all simulations (black), only the 25 simulations with the earliest transmission peak (blue) or the 25 simulations with latest transmission peak (red). Vaccine 1 and 2 result in 50% reduction in symptomatic disease (VE_DIS_) while Vaccine 3 and 4 result in 90% VE_DIS_. Vaccine rollout begins on Dec.1 with 5,000 vaccinated daily till 1,000,000 vaccinations are reached.


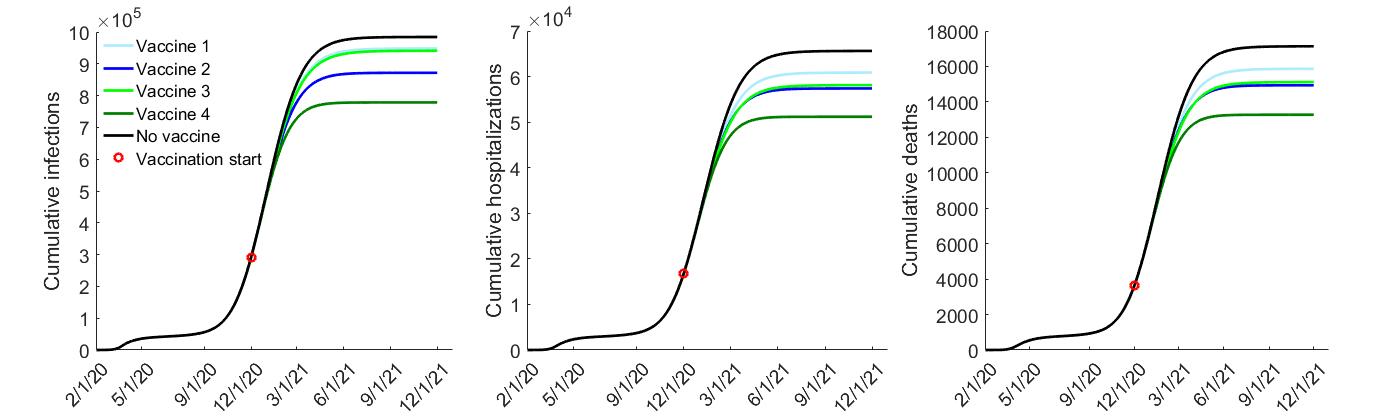


A)

B)

C)

**Figure S7.** **What is the minimum impact to be expected with a licensed vaccine?**  Comparison of: A) cumulative infections; B) cumulative hospitalizations and C) cumulative deaths over time simulated with different efficacy profiles assuming that no asymptomatic and pre-symptomatic cases are diagnosed and the overall infectiousness of individuals who never express symptoms is only 28% lower than symptomatic cases. In all scenarios, the vaccination starts on Dec. 1 and rolled out with 5,000 vaccinated daily till 1,000,000 vaccinations are reached. Vaccine 1 and 2 result in 50% reduction in symptomatic disease (VE_DIS_) while Vaccine 3 and 4 result in 90% VE_DIS_. All projections represent the mean value from 100 epidemic simulations selected at model calibration.

**References**

1 Buitrago-Garcia, D. *et al.* Occurrence and transmission potential of asymptomatic and presymptomatic SARS-CoV-2 infections: A living systematic review and meta-analysis. *PLOS Medicine* **17**, e1003346, doi:10.1371/journal.pmed.1003346 (2020).

2 Centers for Disease Control and Prevention. COVID-19 Pandemic Planning Scenarios. <<https://www.cdc.gov/coronavirus/2019-ncov/hcp/planning-scenarios.html#box>>.

3 Mossong, J. *et al.* Social Contacts and Mixing Patterns Relevant to the Spread of Infectious Diseases. *PLOS Medicine* **5**, e74, doi:10.1371/journal.pmed.0050074 (2008).

4 Bedford, T. *et al.* Cryptic transmission of SARS-CoV-2 in Washington State. *medRxiv*, 2020.2004.2002.20051417 (2020).

5 Thakkar, N. & Famulare, M. COVID-19 transmission was likely rising through April 22 across Washington State. *Institute for Disease Modeling* (2020). <<https://covid.idmod.org/#/ResearchandReports>>.

6 Silva, C. Americans Could See A Vaccine By Mid-December, Says Operation Warp Speed Adviser. (2020). <<https://www.npr.org/sections/coronavirus-live-updates/2020/11/22/937780366/americans-could-see-a-vaccine-by-mid-december-says-operation-warp-speed-adviser>>.
